# Protocol to synthesize the auxin analog 5-Ph-IAA for conditional protein depletion in *C. elegans* using the AID2 system

**DOI:** 10.1016/j.xpro.2024.102901

**Published:** 2024-02-19

**Authors:** Surojit Sural, Juan Quintero Botero, Oliver Hobert, Makeda Tekle-Smith

**Affiliations:** 1 Department of Biological Sciences, Howard Hughes Medical Institute, Columbia University, New York, NY 10027, USA; 2 Department of Chemistry, Columbia University, New York, NY 10027, USA

**Keywords:** Chemistry, Developmental biology, Material sciences, Molecular Biology

## Abstract

The auxin-inducible degron (AID) system is a broadly used tool for spatiotemporal and reversible control of protein depletion in multiple experimental model systems. AID2 technology relies on a synthetic ligand, 5-phenyl-indole-3-acetic acid (5-Ph-IAA), for improved specificity and efficiency of protein degradation. Here, we provide a protocol for cost-effective 5-Ph-IAA synthesis utilizing the Suzuki coupling of 5-chloroindole and phenylboronic acid. We describe steps for evaluating the quality of lab-synthesized 5-Ph-IAA using a *C. elegans* AID2 tester strain.

## Highlights

• Suzuki coupling of 5-chloroindole and phenylboronic acid to synthesize 5-phenylindole
• Synthesis of the AID2 synthetic ligand 2-(5-phenyl-1*H*-indol-3-yl)acetic acid or 5-Ph-IAA
• Subjecting a *C. elegans* AID2 tester strain to 5-Ph-IAA treatment on NGM-OP50 plates
• Quantification of the *in vivo* depletion of AID∗-tagged GFP after 5-Ph-IAA treatment

## Abstract (editor highlights)

Publisher’s note: Undertaking any experimental protocol requires adherence to local institutional guidelines for laboratory safety and ethics.

## Graphical abstract

**Figure:**
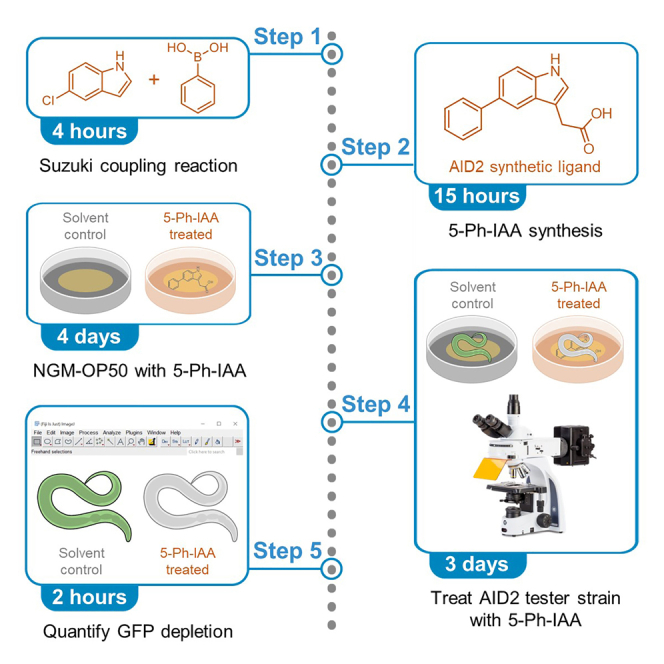

## Before you begin

The auxin-inducible degron (AID) system requires tagging the protein-of-interest with a 44-amino acid degron sequence (termed ‘AID∗’ tag) and the heterologous expression of TIR1, a F-box protein derived from *Arabidopsis thaliana*. In presence of the plant hormone auxin (indole-3-acetic acid, or IAA), which allosterically binds to TIR1, the AID∗-tagged target protein is subjected to degradation via the ubiquitin-proteosome pathway.^1^

Though the AID technology has been applied to multiple experimental systems including yeast, *C. elegans*, *Drosophila*, zebrafish and mammalian cell culture,^1^^,^^2^^,^^3^^,^^4^^,^^5^^,^^6^^,^^7^^,^^8^ it has two major limitations: (1) the wild-type TIR1 protein induces leaky degradation of the AID∗-tagged protein even in the absence of the auxin ligand, IAA, and (2) the millimolar range concentration of IAA required for complete protein depletion generates toxicity and growth retardation in multiple animal models.^9^^,^^10^ A recent improvement of the AID technology, termed AID2, uses a mutated version of the TIR1 protein, TIR1(F79G), which shows undetectable ligand-independent basal activity.^9^^,^^10^^,^^11^ Since the F79G mutation modifies the auxin-binding pocket of TIR1, the AID2 system requires a synthetic derivative of IAA, 5-phenyl-indole-3-acetic acid (5-Ph-IAA), as the ligand for the TIR1(F79G) protein.^9^ In order to achieve similar efficiency of degradation of a target protein, AID2 requires a ∼1,000-fold lower concentration of the 5-Ph-IAA auxin analog in comparison to the doses of natural auxin used in the classical AID system.^9^^,^^10^^,^^11^ Though the AID2 improvement almost completely alleviates both the major limitations, namely leaky degradation and ligand-induced toxicity, of the original AID system, the high cost and limited availability of the synthetic auxin derivative, 5-Ph-IAA, has hindered the broad use of this new technology across experimental labs.

Here we outline a cost-effective protocol to chemically synthesize 5-Ph-IAA in any laboratory that is equipped to perform standard organic synthesis reactions. This protocol uses commonly available low-cost reagents to synthesize 5-Ph-IAA at 4 mmol scale. In addition, we provide detailed steps to evaluate the quality of lab-synthesized 5-Ph-IAA in terms of its ability to induce *in vivo* degradation of an AID∗-tagged protein in an AID2-compatible *Caenorhabditis elegans* animal model.^10^ Standard techniques to grow *C. elegans* in laboratory conditions can be found in WormBook^12^ and protocols to prepare culture plates, bacterial food source and agarose microscopy pads for *C. elegans* has been previously described by Wang et al.^13^ The 5-Ph-IAA compound synthesized using our low-cost protocol should be usable with any experimental system that is adapted to use the AID2 technology, including yeast, mammalian cells and mice.^9^

## Key resources table

**Table:** 

REAGENT or RESOURCE	SOURCE	IDENTIFIER
**Bacterial and virus strains**		

*Escherichia coli* OP50 strain	Caenorhabditis Genetics Center	https://cgc.umn.edu/strain/OP50

**Chemicals, peptides, and recombinant proteins**		

Palladium(II) acetate	Sigma-Aldrich	Catalog # 205869
XPhos	Sigma-Aldrich	Catalog # 638064
5-Chloroindole	Sigma-Aldrich	Catalog # C47604
Phenylboronic acid	Sigma-Aldrich	Catalog # P20009
Cesium hydroxide hydrate	Sigma-Aldrich	Catalog # C8518
Oxalyl chloride	Sigma-Aldrich	Catalog # 221015
2-Ethoxyethanol	Sigma-Aldrich	Catalog # 128082
Sodium methoxide	TCI Chemicals	Catalog # S0485
Hydrazine monohydrate	Sigma-Aldrich	Catalog # 207942
Ethyl alcohol	Sigma-Aldrich	Catalog # E7023
Granulated agar	BD Difco	Catalog # 214530
Tryptone	Fisher Scientific	Gibco 211705
Yeast extract	Fisher Scientific	Gibco 212750
Peptone	Fisher Scientific	Gibco 211677
Sodium hypochlorite solution	Sigma-Aldrich	Catalog # 239305
5-phenyl-indole-3-acetic acid (5-Ph-IAA)	BioAcademia	Catalog # 30-003-10

**Experimental models: Organisms/strains**		

*Caenorhabditis elegans* HML1012 strain: *cshIs140[rps-28p::TIR1(F79G)::T2A::mCherry::his-11 + Cbr-unc-119(+)] II; ieSi58 [eft-3p::degron::GFP::unc-54 3′UTR + Cbr-unc-119(+)] IV*	Caenorhabditis Genetics Center	RRID: WB-STRAIN:WBStrain00050713
*Caenorhabditis elegans* wild-type N2 strain	Caenorhabditis Genetics Center	RRID: WB-STRAIN:WBStrain00000001

**Software and algorithms**		

Fiji image analysis software	Schindelin et al.^14^	https://imagej.net/software/fiji

**Other**		

60 mm polystyrene Petri dish	Tritech Research	Catalog # T3308
1.5 mL black micro centrifuge tube	CELLTREAT Scientific	Catalog # 229437
1.5 mL natural micro centrifuge tube	USA Scientific	Catalog # 1615-5510
Millex-GP 0.22 μm filter	Millipore	Catalog # SLGP033RS

## Materials and equipment

**Table:** LB agar plates

Reagent	Final concentration	Amount
Agar (powder)	15 mg/mL	15 g
Tryptone (powder)	10 mg/mL	10 g
Yeast extract (powder)	5 mg/mL	5 g
NaCl (powder)	10 mg/mL	10 g
ddH_2_O	N/A	up to 1 L
**Total**	**N/A**	**1 L**

Sterilize by autoclaving. Cool to 55°C and pour 30 mL of NGM per 100 mm petri dish and let solidify at room temperature (20°C). LB agar plates can be stored at 4°C for up to two weeks.

**Table:** LB liquid medium

Reagent	Final concentration	Amount
Tryptone (powder)	10 mg/mL	10 g
Yeast extract (powder)	5 mg/mL	5 g
NaCl (powder)	10 mg/mL	10 g
ddH_2_O	N/A	up to 1 L
**Total**	**N/A**	**1 L**

Sterilize by autoclaving. Cool to room temperature (20°C). Can be stored at room temperature (20°C) for up to 4 months.

**Table:** Nematode Growth Medium (NGM)

Reagent	Final concentration	Amount
Agar (powder)	19 mg/mL	19 g
Peptone (powder)	2.5 mg/mL	2.5 g
NaCl (powder)	3 mg/mL	3 g
ddH_2_O	N/A	972 mL
Sterilize by autoclaving. Cool to 55°C with constant stirring. Then add the following ingredients:		
KH_2_PO_4_ solution (1 M, pH 6.0)	25 mM	25 mL
MgSO_4_ solution (1 M)	1 mM	1 mL
CaCl_2_ (1 M)	1 mM	1 mL
Cholesterol (5 mg/mL in ethanol)	5 μg/mL	1 mL
**Total**	**N/A**	**1 L**

Pour 10 mL of NGM per 60 mm petri dish and let solidify at room temperature (20°C). NGM plates can be stored at 4°C for up to one month.

***Alternatives:*** If fungal contamination of NGM plates is a concern, the anti-fungal compound nystatin can be added to NGM at a final concentration of 2.5 μg/mL after autoclaving and cooling the NGM to 55°C.

**Table:** M9 buffer

Reagent	Final concentration	Amount
Na_2_HPO_4_ (powder)	6 mg/mL	6 g
KH_2_PO_4_ (powder)	3 mg/mL	3 g
NaCl (powder)	5 mg/mL	5 g
ddH_2_O	N/A	999 mL
Sterilize by autoclaving. Cool to room temperature (20°C). Then add the following:		
MgSO_4_ solution (1 M)	1 mM	1 mL
**Total**	**N/A**	**1 L**

Can be stored at room temperature (20°C) for up to 3 months.

**Table:** Alkaline bleaching solution

Reagent	Final concentration	Amount
Sodium hypochlorite solution (5%)	1%	2 mL
NaOH solution (5 M)	500 mM	1 mL
ddH_2_O	N/A	7 mL
**Total**	**N/A**	**10 mL**

Prepare fresh every time before use. If needed, can be stored protected from light at 4°C for up to a week.

## Step-by-step method details

### Suzuki coupling of 5-chloroindole and phenylboronic acid

**Timing: 4 h**

**Figure 1:**
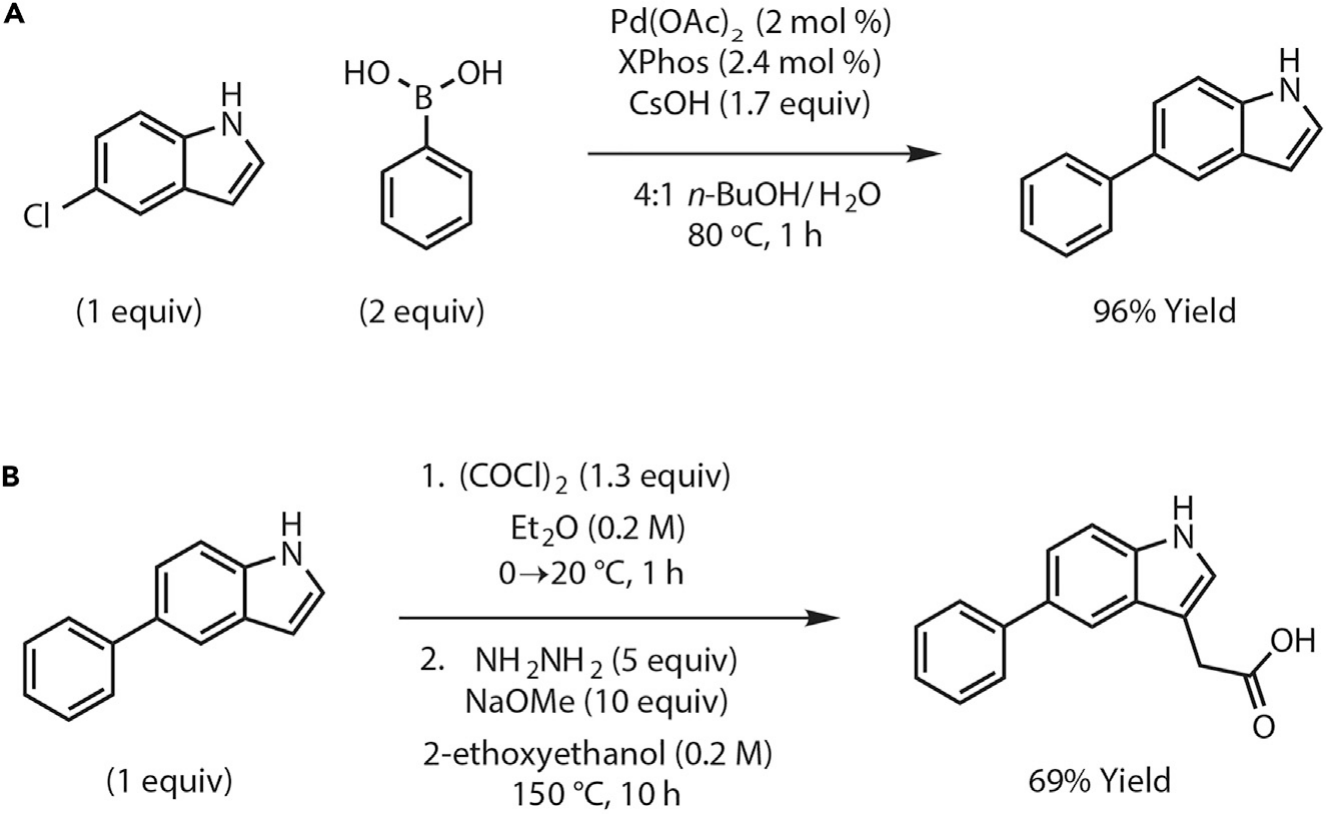
Chemical synthesis of the auxin analog 5-Ph-IAA (A) Suzuki coupling of 5-chloroindole and phenylboronic acid. (B) Synthesis of 2-(5-phenyl-1*H*-indol-3-yl)acetic acid or 5-Ph-IAA from 5-phenylindole.

This step performs a Suzuki coupling reaction of 5-chloroindole and phenylboronic acid to generate the compound 5-phenylindole (Figure 1A).^15^

1. Sequentially add to a flame-dried 40 mL vial, equipped with a Teflon stir bar, Pd(OAc)_2_ (2.0 mol %, 0.011 mmol, 2.1 mg), XPhos (2.4 mol %, 0.01 mmol, 6.2 mg), 5-chloroindole (1.0 equiv, 0.54 mmol, 83 mg) and phenylboronic acid (2.0 equiv, 1.1 mmol, 133 mg) at room temperature (20°C).
2. Once reagents from Step 1 are added to the vial, evacuate the vial for 30 s and then refill the reaction vial with nitrogen. Repeat this process two more times.
3. Introduce 3.0 mL of degassed *n*-butanol into the reaction vial using a syringe under a nitrogen atmosphere.
4. Add CsOH (1.7 equiv, 0.93 mmol, 155 mg) to a separate flame-dried vial equipped with a Teflon stir bar.
5. Evacuate the vial for 30 s and then refill this vial with nitrogen. Repeat this process two more times.
6. Add 763 μL of degassed water to the CsOH-containing vial and stir the mixture until complete dissolution of CsOH.
7. Add the resulting hydroxide solution to the reaction mixture (from step 3) dropwise via a syringe.
8. Submerge the reaction setup in an oil bath and heat to 80°C. Allow the reaction to proceed for 1 h under a nitrogen atmosphere. Refer to troubleshooting 1.
9. After 1 h, allow the reaction mixture to reach room temperature (20°C) by removing the oil bath heating source and then transfer into a 250 mL separatory funnel.
10. Extract the reaction mixture that was transferred to the 250 mL separatory funnel in step 9 with ethyl acetate (3 × 40 mL).
11. Combine the organic layers into a single flask, dry with sodium sulfate (add sodium sulfate until particulates float freely without clumping when swirled) and subsequently vacuum filter.
12. Concentrate the resulting solution using a rotary evaporator to obtain a pale yellow oil.
13. Obtain a ^1^H NMR of the crude material to confirm product formation.
14. Remove excess *n*-butanol prior to purification via azeotropic vacuum distillation with toluene.
15. Purify the crude product by silica gel flash (230–400 mesh) column chromatography (0:100 → 15:85 EtOAc:Hexanes) to obtain 5-phenylindole (95.7 mg, 96% yield) as a white solid.
16. Obtain a ^1^H NMR, ^13^C NMR and HRMS of the purified product to confirm identity and purity.

### Synthesis of 2-(5-phenyl-1*H*-indol-3-yl)acetic acid (or 5-Ph-IAA)

**Timing: 15 h**

This step describes the synthesis of 2-(5-phenyl-1*H*-indol-3-yl)acetic acid (or 5-Ph-IAA) from 5-phenylindole via a 2-oxo-2-(5-phenyl-1*H*-indol-3-yl)acetic acid intermediate (Figure 1B).^16^

17. Prepare a 0.2 M solution of 5-phenylindole (1.0 equiv, 5.9 mmol, 1.14 g) (from step 15) in diethyl ether (30 mL) in a 200 mL round bottom flask. Cool the resulting solution to 0°C in an ice water bath.
18. Slowly add oxalyl chloride (1.3 equiv, 7.7 mmol, 658 μL) to the 5-phenylindole solution at 0°C with constant stirring. Stir the reaction mixture for 1 h at room temperature (20°C).
19. Precipitation of 2-oxo-2-(5-phenyl-1*H*-indol-3-yl)acetic acid.
  a. Add a mixture of diethyl ether and water (v/v = 2:1, 26 mL) to the reaction mixture from step 18 at 0°C.
  b. Collect the resulting yellow precipitate of 2-oxo-2-(5-phenyl-1*H*-indol-3-yl)acetic acid by filtration.
  c. Use this compound directly for the next step in the synthetic sequence.
20. Dissolve 2-oxo-2-(5-phenyl-1*H*-indol-3-yl)acetic acid (1 equiv, 5.9 mmol, 1.57 g) in 30 mL of 0.2 2-ethoxyethanol.
21. Sequentially add 3.19 g of sodium methoxide (10.0 equiv, 59.0 mmol) and 1.43 mL of hydrazine monohydrate (5.0 equiv, 29.5 mmol) to the above solution at room temperature (20°C).
22. Stir the resulting mixture at 150°C for 10 h in a 75 mL pressure vessel flask.

**CRITICAL:** The reaction should be heated and stirred behind a blast shield during this 10 h reaction.

23. Allow the reaction mixture to reach room temperature (20°C) by removing the heat source and subsequently dilute it with water until complete dissolution.
24. Acidify the reaction mixture to maintain a pH below 4.0 using 2 M aqueous hydrochloric acid.
25. Filtration and concentration of 5-Ph-IAA as a crude product.
  a. Extract the aqueous solution from step 24 three times with ethyl acetate using a separatory funnel.
  b. Dry the organic layer over Na_2_SO_4_ (add sodium sulfate until particulates float freely without clumping when swirled).
  c. Filter and concentrate under reduced pressure, with toluene serving as an azeotrope.
26. Purify the crude product with silica gel flash column chromatography (0:100 → 15:85 MeOH:chloroform) to produce 5-Ph-IAA as a light orange oil.
27. Subject this oil to azeotropic distillation with toluene to eliminate any residual 2-ethoxyethanol.
28. Collect 5-Ph-IAA as a white powder (1.02 g, 69% yield over 2 steps) and store in a sterile and clean glass container at 4°C for > 2 years. Refer to troubleshooting 2.
29. Obtain identity and purity of 5-Ph-1AA via ^1^H NMR, ^13^C NMR, and HRMS.

**CRITICAL:** 5-Ph-IAA is light-sensitive and hence, for long-term storage at 4°C, the container with 5-Ph-IAA should be wrapped in aluminum foil to minimize exposure to ambient light. The cap of the container should be wrapped with a layer of parafilm to prevent moisture from getting inside the container.

### Preparing NGM-OP50 plates with the desired dose of 5-Ph-IAA

**Timing: 4 days**

These steps outline the protocol to prepare nematode culturing plates with the desired concentration of 5-Ph-IAA.

30. Preparing a 100 mM stock solution of 5-Ph-IAA:
  a. Using an analytical balance, weight approximately 100 mg of 5-Ph-IAA powder and transfer to a 15 mL conical tube.
  b. To prepare a 100 mM stock of 5-Ph-IAA (molecular weight: 251.29 g/mol), add 3.98 mL of 96% ethanol to the tube and wrap the tube with aluminum foil to protect it from light.
    ***Note:*** If a different weight of 5-Ph-IAA is used, the volume of ethanol should be adjusted accordingly.
  c. Vigorously vortex the tube for 20 s and then put it on a nutating shaker at room temperature (20°C) for 10 min.
  d. After 10 min, check if the 5-Ph-IAA powder has completely dissolved in the solvent. If not, vortex the tube again for 20 s, put it on the shaker and monitor at 5 min intervals till the powder dissolves completely.
    ***Note:*** On complete dissolution, the 100 mM stock of 5-Ph-IAA will be light brown in color.
  e. Prepare 500 μL aliquots of the 100 mM 5-Ph-IAA solution in black 1.5 mL microcentrifuge tubes (to protect the solution from light) and store them at −80°C for up to 1 year.
    ***Note:*** If black 1.5 mL microcentrifuge tubes are not available, the 100 mM 5-Ph-IAA solution can be aliquoted in clear 1.5 mL microcentrifuge tubes that are individually wrapped with aluminum foil.
    **CRITICAL:** 5-Ph-IAA has limited solubility in water and hence, we recommend dissolving 5-Ph-IAA in 96% ethanol, as described in a previous study.^10^ As mentioned in step 33c, approximately 100 μL of the solvent is needed to cover the entire surface of a 60 mm diameter NGM-OP50 agar plate, which results in 1% (v/v) final concentration of the solvent in agar, i.e., 100 μL solvent in 10 mL agar. We allow the ethanol to evaporate from the NGM-OP50 plates for at least 16 h (detailed below) and the final ethanol concentration on the plates (∼100 mM) is much lower than doses at which ethanol induces behavioral or developmental phenotypes in *C. elegans*.^17^^,^^18^ Some studies have alternatively used dimethyl sulfoxide (DMSO) as a solvent to dissolve 5-Ph-IAA.^9^^,^^11^ Though DMSO is generally considered harmless at low concentrations for most organisms, 1% DMSO has been demonstrated to significantly alter feeding behavior and activate stress response pathways in *C. elegans*.^19^^,^^20^ If the experimenter intends to prepare the 100 mM stock solution of 5-Ph-IAA in DMSO, the working solution of 100 μM 5-Ph-IAA (described in step 33a) should be prepared in a 1:10 dilution of DMSO in M9 buffer, which makes the effective DMSO concentration 0.1% at which it does not produce behavioral changes in *C. elegans*.^20^ If any other organic solvent is used to dissolve 5-Ph-IAA, ideally the solvent itself should not have any physiological or behavioral effects on the organism being studied. For all experiments, the effects of 5-Ph-IAA should always be evaluated in comparison with control animals treated with solvent alone.
31. Preparing bacterial food in LB liquid medium:
  a. Using sterile technique, use a 10 μL pipette tip to pick a single colony of *E. coli* OP50 strain from an LB agar plate and inoculate in a bottle containing 500 mL of sterile LB liquid medium.
  b. Put the bottle in a 37°C incubator for 12–14 h.

***Note:*** This bacterial food can be used immediately or can be stored at 4°C for up to 2 weeks.

32. Growing OP50 bacteria on NGM plates:
  a. Make sure that NGM plates are warmed to room temperature (20°C).
  b. If the OP50 bacteria in LB liquid medium were stored at 4°C, shake the bottle in circular motion to mix the bacteria uniformly in case it has settled at the bottom of the bottle.
  c. Under sterile conditions, pipette 100 μL of bacteria on top of each 60 mm NGM plate and immediately spread the liquid to create a uniform lawn of bacteria on the surface of the NGM plate.
  d. Prepare the required number of such NGM-OP50 lawn plates (at least two plates per condition).
  e. Let the bacterial lawn dry at room temperature (20°C) with lid closed for 16–20 h.
33. Adding 5-Ph-IAA to NGM-OP50 plates:
  a. In a black 1.5 mL microcentrifuge tube, add 1 μL of 100 mM 5-Ph-IAA stock solution and 999 μL of 96% ethanol.
    ***Note:*** If black 1.5 mL microcentrifuge tubes are not available, a clear 1.5 mL microcentrifuge tube wrapped with aluminum foil can be used as an alternative.
  b. Mix by pipetting several times. The resulting concentration of 5-Ph-IAA solution will be 100 μM.
  c. Pipette 100 μL of the 100 μM 5-Ph-IAA solution on top of each NGM-OP50 lawn plate and swirl the plate so that the solution covers the entire surface of the OP50 lawn.
  d. Immediately transfer the plates to an opaque box to protect the 5-Ph-IAA from light. The final concentration of 5-Ph-IAA in the NGM plate is expected to be 1 μM.
  e. Prepare the required number of 1 μM 5-Ph-IAA NGM-OP50 plates (at least two plates per condition) and protect all plates from light.
  f. For control plates, add 100 μL of 96% ethanol on top of each NGM-OP50 lawn plate and similarly, swirl the plate to cover the entire surface of the OP50 lawn.
  g. Prepare at least two plates for each condition.
  h. If a wild-type strain not expressing any fluorescent protein will be used, prepare the required number of ethanol only plates for that strain.
  i. Store both 1 μM 5-Ph-IAA and ethanol only plates in the dark for 16–20 h and use immediately after that.
    ***Note:*** We do not recommend storage of NGM plates with 5-Ph-IAA for longer than a week since the effective concentration of the compound might reduce over time and can make effect sizes variable across independent trails of the experiment.

### Subjecting the *C. elegans* AID2 tester strain to 5-Ph-IAA treatment

**Timing: 3 days**

In this step, the AID2 tester strain, HML1012, will be subjected to 5-Ph-IAA treatment from early development till adulthood to deplete the AID∗-tagged GFP protein.

34. Grow the *C. elegans* strains to be used in the experiment.
  a. Culture the *C. elegans* HML1012 strain in 60 mm NGM plates seeded with OP50 bacteria using standard worm maintenance technique.^12^
  b. If a non-transgenic control is needed, also grow the N2 strain in similar conditions and follow the subsequent steps for all strains in parallel.
35. Wash gravid adult worms from a crowded (but non-starved) plate of the HML1012 strain using 1.6 mL of M9 buffer and collect the solution in a 1.5 mL centrifuge tube.
36. Centrifuge the tube at 300 × *g* for 1 min.
37. Carefully remove as much M9 buffer as possible without disturbing the worm pellet at the bottom of the tube.
38. In a 15 mL conical tube, prepare 10 mL of alkaline bleaching solution (see Materials for recipe).

***Note:*** The alkaline bleaching solution should always be freshly prepared and should be used within 15 min of preparing the solution.

39. Fill the 1.5 mL microcentrifuge tube containing the worm pellet with alkaline bleaching solution and store the tube horizontally on the bench.
40. Vigorously shake the tube every 30 s and check it under a dissecting microscope to see if the bodies of the adult worms have dissociated.

**CRITICAL:** Do not let the embryos remain in alkaline bleaching solution for more than 5 min.

41. As soon as the bodies of the adult worms have dissociated and unhatched embryos from inside the adults have been released in the solution, immediately centrifuge the tube at 1,300 × *g* for 1 min.
42. Carefully remove as much alkaline bleaching solution as possible without disturbing the embryos at the bottom of the tube.
43. Wash the embryos with M9 buffer.
  a. Fill the tube with M9 buffer and centrifuge at 1,300 × *g* for 1 min.
  b. Carefully remove as much M9 buffer as possible.
44. Repeat M9 washes 2 additional times with centrifugation at 1,300 × *g* for 1 min for each washing step.
45. Carefully remove all M9 buffer from the washes and resuspend the unhatched embryos in 500 μL of M9 buffer.
46. Resuspend the embryos by pipetting up and down several times.

**CRITICAL:***C. elegans* embryos in buffer will settle to the bottom the tube within 30 s if not resuspended properly by pipetting up and down.

47. Determine the density of embryos in the suspension.
  a. Pipette 20 μL of the solution from step 46 on a glass slide and count the number of embryos by observing under a dissecting microscope.
  b. Estimate the total number of embryos in the 500 μL suspension.
48. Transfer embryos to NGM-OP50 plates.
  a. Mix the embryo suspension by pipetting up and down several times and transfer ∼100 HML1012 strain embryos per plate to two NGM-OP50 plates with 1 μM 5-Ph-IAA and two control NGM-OP50 plates with ethanol alone.
  b. If the wild-type N2 strain is used in the experiment, transfer ∼100 N2 strain embryos per plate to two NGM-OP50 plates with ethanol alone.

***Note:*** If less than 25 animals per condition are needed for the experiment, steps 35 to 48 can be skipped, and instead, transfer 10 gravid adult worms of the HML1012 strain using a platinum pick to two NGM-OP50 plates with 1 μM 5-Ph-IAA and to two control NGM-OP50 plates with ethanol alone. For the wild-type N2 strain, transfer 10 gravid adults per plate to two NGM-OP50 plates with ethanol alone. Put all the plates in a dark box in a 25°C incubator and let the adults lay eggs on the plates for 2 h. After 2 h, pick and remove all the adults from the plates using a platinum pick and proceed to step 49.

49. Put all plates in a 25°C incubator for 48 h. Place the plates inside a dark box to protect them from light.

**CRITICAL:** If the *C. elegans* strain used is temperature-sensitive, put the strain at the required temperature. The time taken for development to adulthood should be monitored if a different growth temperature is used.

50. After 48 h, observe the plates under a dissecting scope to confirm that all the worms are synchronized as young adults. Refer to troubleshooting 3.
51. Preparing a 50 mM stock solution of sodium azide.
  a. Prepare a 10 mL solution of 50 mM sodium azide in M9 buffer.
  b. Filter sterilize this solution using a 0.22 μm filter.

***Note:*** The filter-sterilized solution of sodium azide can be stored at room temperature (20°C) for up to a year.

52. Prepare an agarose pad on top of a glass slide using the protocol described in Wang et al.^13^
  a. Put a layer of adhesive tape of ∼200 μm thickness on two glass slides.
  b. Put a clean glass slide between these two slides, with all three slides aligned in parallel.
  c. Place ∼100 μL of 2% molten agarose in M9 buffer on the clean slide in the middle.
  d. Immediately place another clean glass slide orthogonally on top of the agarose drop to flatten it between the two clean glass slides.
  e. After 1 min, carefully separate the slides so that the flattened solidified agarose pad remains attached to one slide.
53. Add 6 μL of the 50 mM sodium azide solution on top of the agarose pad.
54. Transfer worms to the agarose pad.
  a. Using a platinum pick, transfer ∼10–15 young adult worms into the sodium azide solution on the agarose pad.
  b. Use the platinum pick to gently separate the worms if they are clumped together in aggregates.

***Note:*** The worms should be anesthetized by sodium azide in 1–2 min.

**Figure 2:**
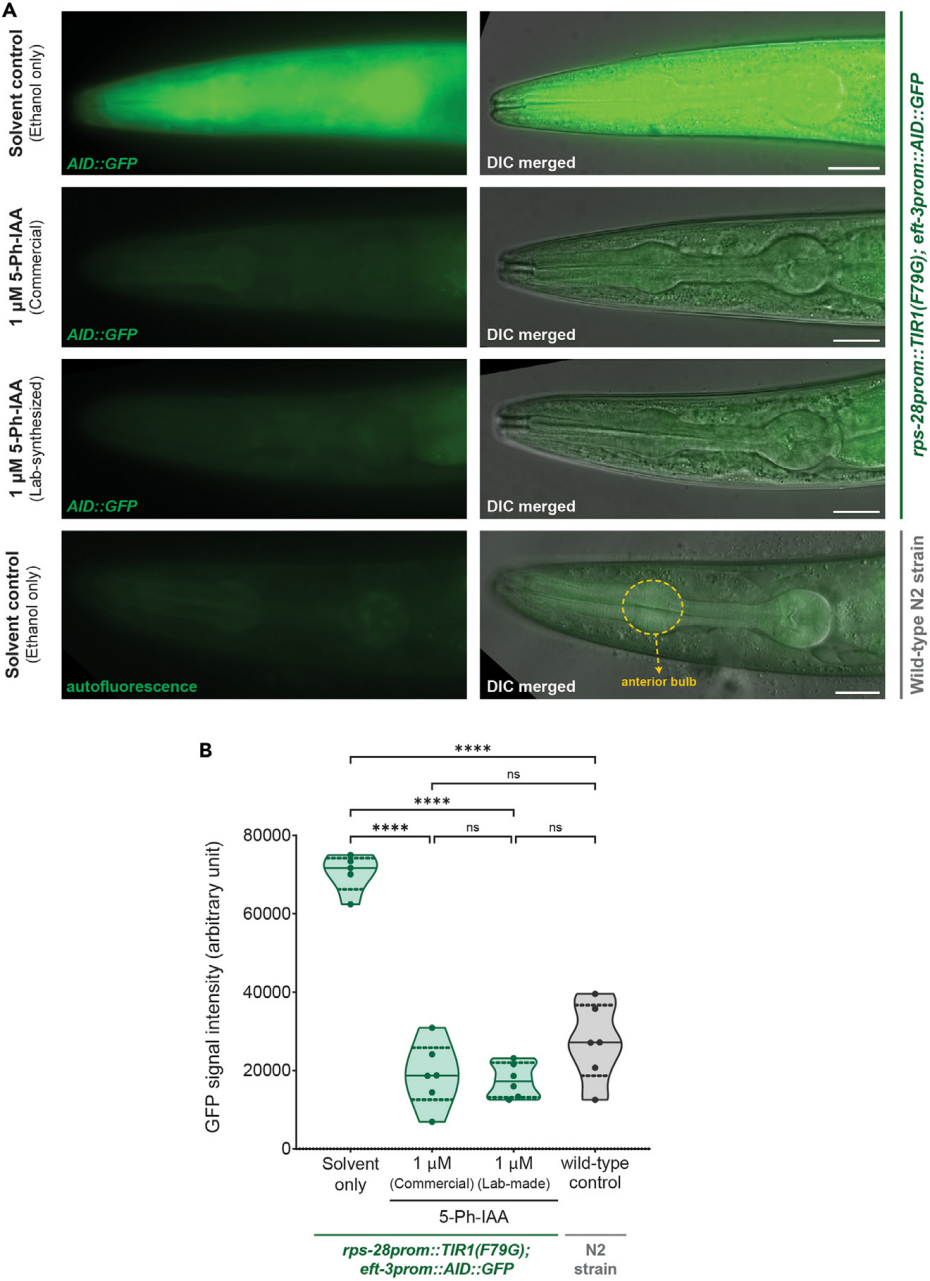
Testing the efficiency of degradation of AID∗-tagged GFP protein using lab-synthesized 5-Ph-IAA (A) Images showing heads of adult animals of the HML1012 strain grown in the presence of 1 μM 5-Ph-IAA (either commercial or lab-synthesized) or ethanol alone. The head of an adult of the wild-type N2 strain is also shown for estimation of autofluorescence from the GFP channel. The location of the anterior bulb of the pharynx is shown for the wild-type strain. Scale bars, 20 μm. (B) Violin plots for GFP intensity from the anterior bulb of the pharynx in conditions shown in (A). Median and interquartile range are shown as horizontal lines inside the violin distribution for 5–6 animals per condition. ∗∗∗∗ and ‘ns’ indicate p < 0.0001 and no significant difference, respectively, in Tukey’s multiple comparisons test performed after one-way ANOVA.
  b. For each animal, capture multiple images across a z-stack with an interval of 1 μm between slices to cover the entire thickness of the worm.
  c. Repeat for a total of at least 5–6 worms per condition and image both 5-Ph-IAA and control conditions on the same day. Refer to troubleshooting 4.

***Note:*** For capturing GFP images, select an excitation intensity and exposure time such that the captured GFP signal is within the linear range of detection and not overexposed. Use the same settings to capture all images across different conditions.

55. Gently place a glass cover slip on top of the worms.
56. Put the slide under a fluorescent microscope that is equipped with capturing GFP signal (excitation wavelength range: 460–490 nm, emission wavelength range: 500–540 nm).
57. Capture images of the worms on the microscope.
  a. Using a 40× objective, focus on the head region of a worm to capture images at the GFP and DIC channels (Figure 2A).

### Quantification of the depletion of AID∗-tagged GFP after 5-Ph-IAA treatment

**Timing: 2 h**

This step quantifies GFP signal intensity across control and 5-Ph-IAA-treated conditions to estimate the extent of depletion of AID∗-tagged GFP protein.

58. Open the Fiji software on a computer.^14^
59. Import an image of the head of an adult worm of the HML1012 strain grown in NGM-OP50 with ethanol alone.
60. Open the GFP channel of the image and covert it to 8-bit format by selecting *Image > Type > 8-bit* (unless it is already in that format).
61. Go to the DIC channel and select the z-slice that has the anterior bulb of the pharynx in focus (an example shown in Figure 2A).
62. Open the corresponding z-slice of the GFP channel and draw an outline of the anterior bulb of the pharynx using the ‘*Freehand selections’* tool of Fiji.

***Note:*** We recommend quantifying the GFP signal from the anterior bulb of the pharynx because its anatomical location is easy to locate in the head region of the animal. The GFP intensity from another body part can also be quantified as long as autofluorescence from the intestine does not interfere with that signal.

63. Click *Analyze > Measure.*

***Note:*** This will output multiple values corresponding to the signal intensity of the outlined region of the anterior bulb.

64. Copy the *Area*, *Mean* and *Integrated Density* values to an excel spreadsheet.

***Note:****Integrated Density* refers to the total GFP signal intensity within the outlined anterior bulb.

65. On the same z-slice of the GFP channel, outline a region outside the worm using the ‘*Freehand selections’* tool to capture the background signal intensity.

***Note:*** The area of this background region does not need to be equal to the size of the anterior bulb.

66. Click *Analyze > Measure* and copy the *Area*, *Mean* and *Integrated Density* values of the background region to an excel spreadsheet.
67. Calculate the background subtracted fluorescence intensity.
  a. Multiply the *area* value of the anterior bulb to the *mean* value of background region.
    ***Note:*** This will calculate the expected background signal from the anterior bulb.
  b. Subtract this value from the *Integrated Density* value of the anterior bulb to obtain the background subtracted fluorescence intensity from the anterior bulb as shown in the formula below.
    Backgroundsubtractedfluorescenceintensity=Integrateddensityofanteriorbulb(step64)−[Areaofanteriorbulb(step64)XMeanintensityvalueofbackgroundregion(step66)]
68. Using the above steps, calculate the background subtracted fluorescence intensity from the anterior bulb in all the acquired images for all conditions.
69. Perform statistical analysis to compare the GFP signal intensity across the different conditions (Figure 2B). Refer to troubleshooting 5.

## Expected outcomes

**Figure 3:**
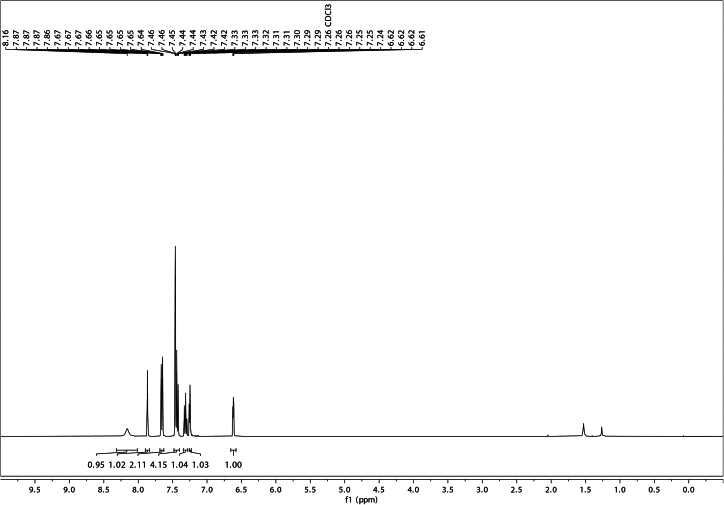
^1^H NMR of 5-phenylindole in CDCl_3_

**Figure 4:**
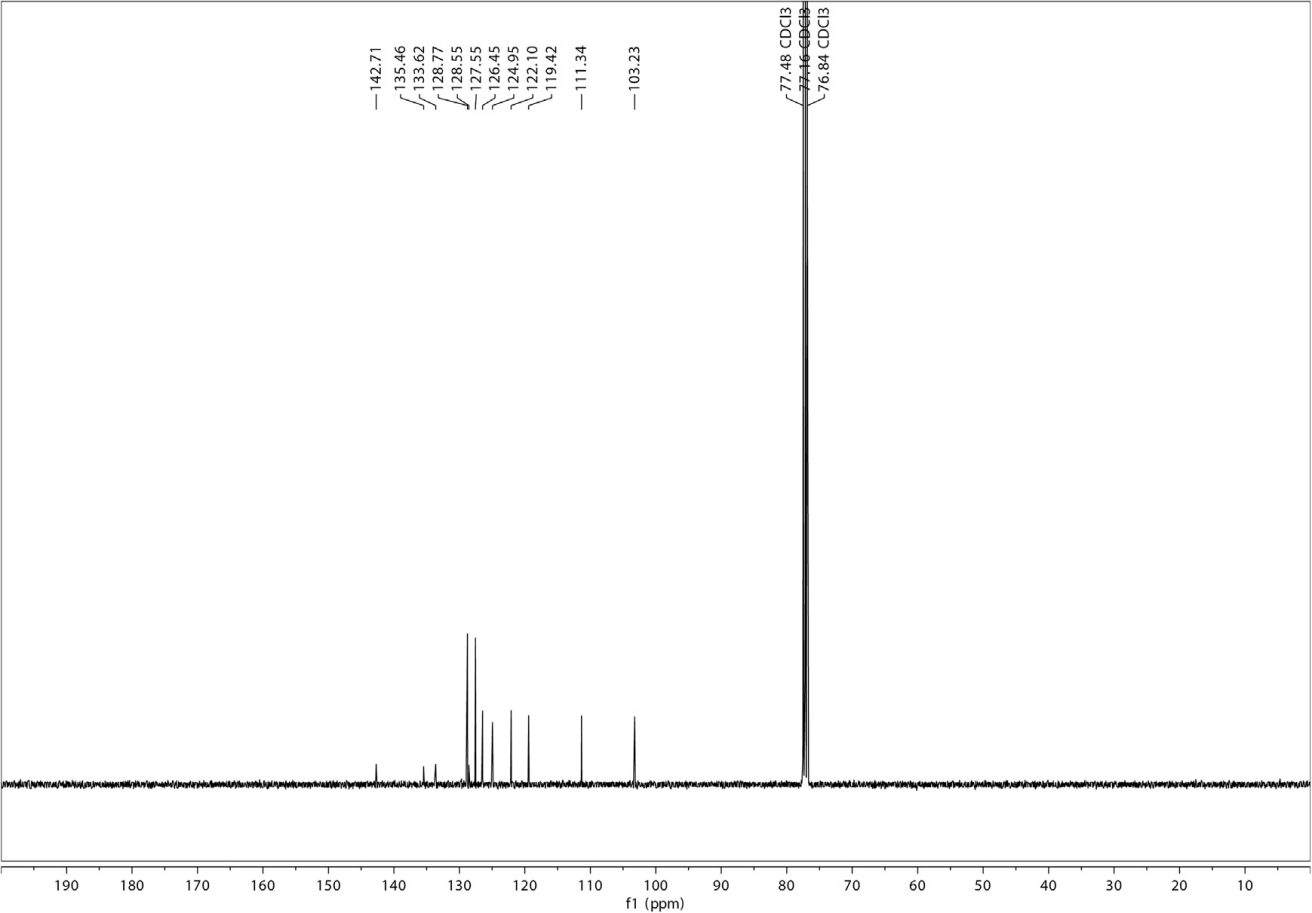
^13^C NMR of 5-phenylindole in CDCl_3_

Using the described chemical synthesis protocol, Suzuki coupling of 5-chloroindole and phenylboronic acid should generate 5-phenylindole of very high purity (Figures 3 and 4). Characterization data for 5-phenylindole:

^1^H NMR (400 MHz, CDCl_3_) δ 8.16 (s, 1H), 7.87–7.86 (m, 1H), 7.68–7.63 (m, 2H), 7.48–7.40 (m, 4H), 7.34–7.28 (m, 1H), 7.25 (dd, J = 3.3, 2.4 Hz, 1H), 6.62 (dd, J = 3.3, 2.0 Hz, 1H).

^13^C NMR (101 MHz, CDCl_3_) δ 142.7, 135.5, 133.6, 128.8, 128.6, 127.6, 126.5, 124.9, 122.1, 119.4, 111.3, 103.2.

HRMS (ESI+) calculated m/z C_14_H_12_N [M + H]^+^: 194.0970, found 194.0967.

**Figure 5:**
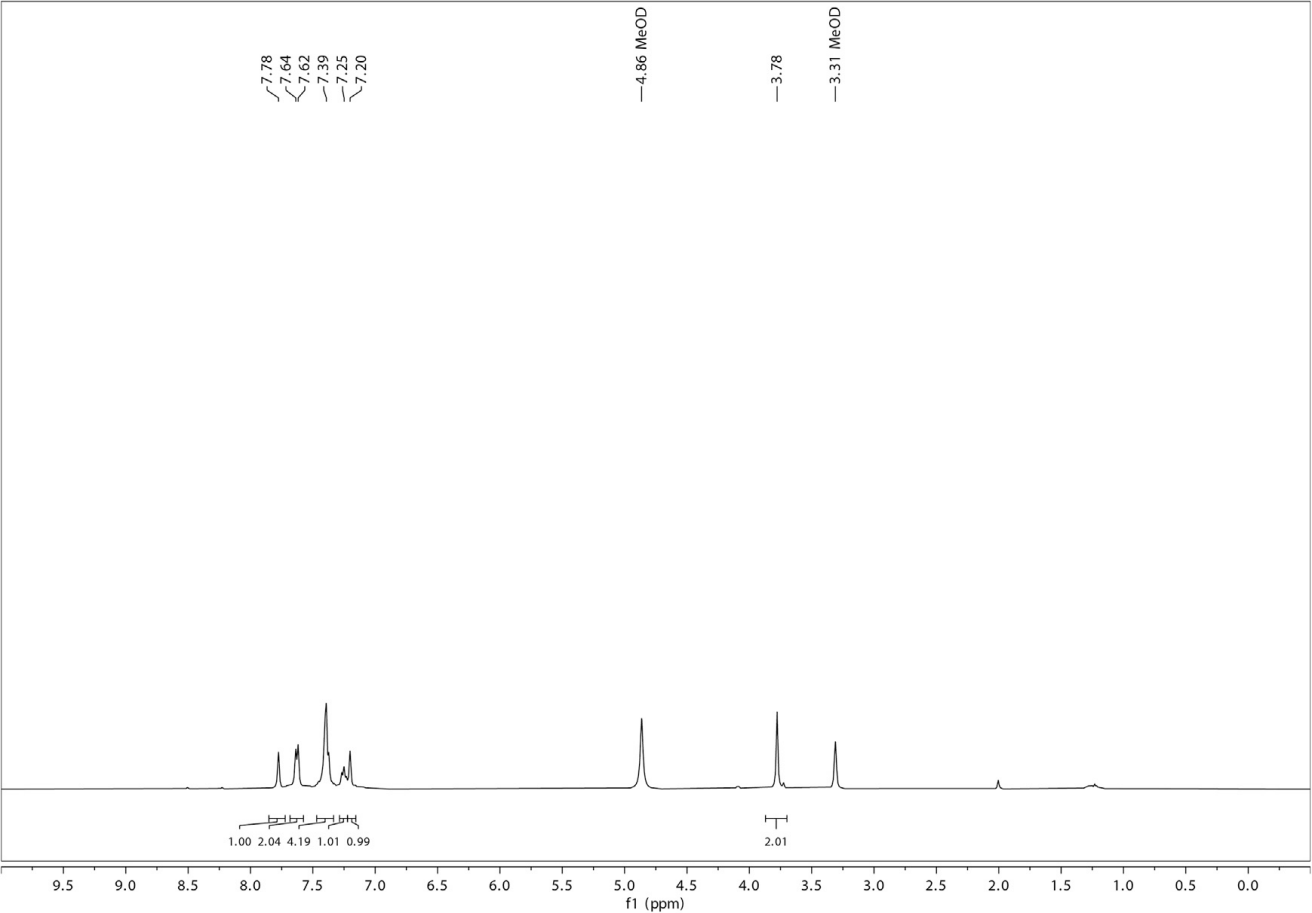
^1^H NMR of 5-Ph-IAA in CD_3_OD

**Figure 6:**
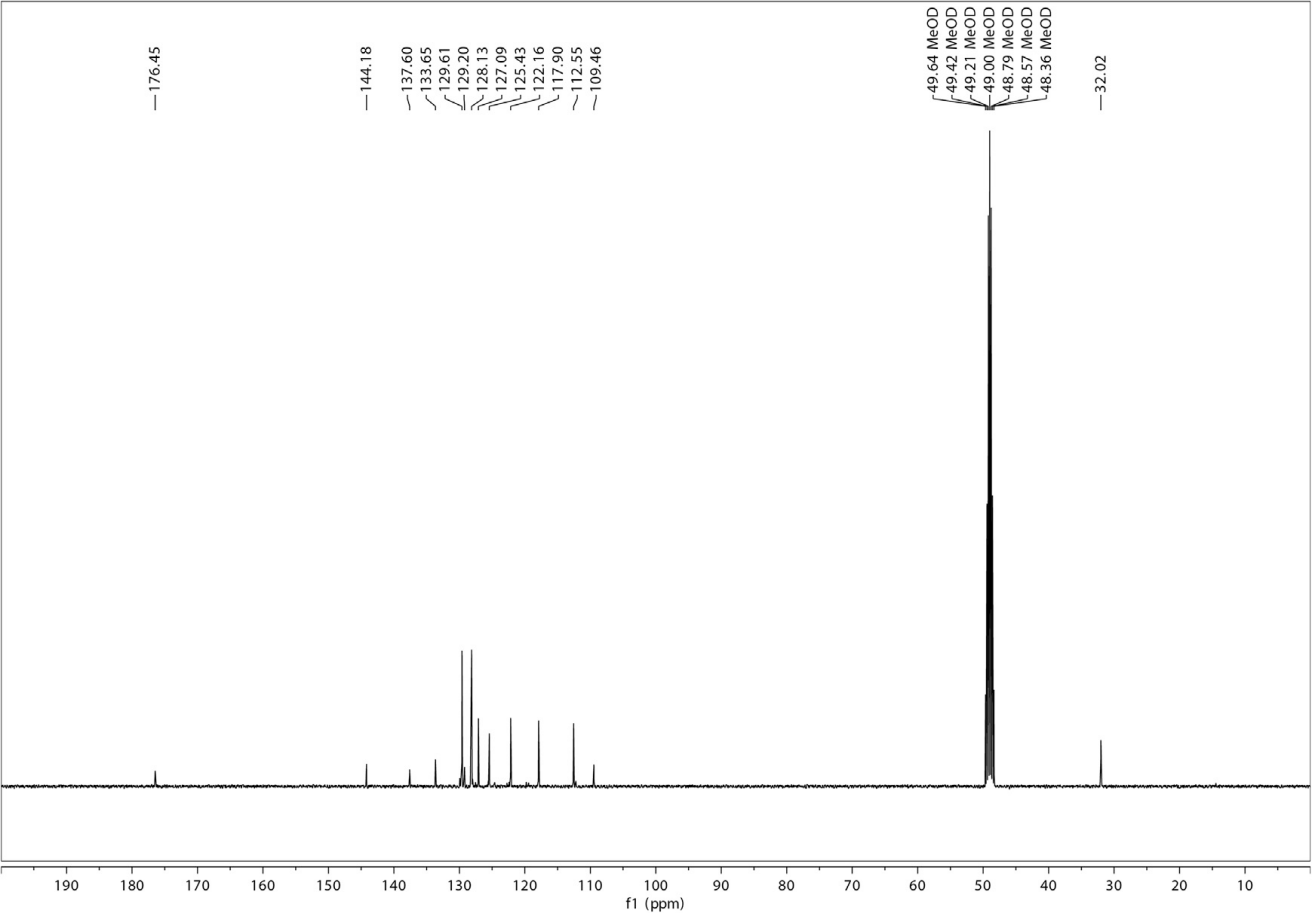
^13^C NMR of 5-Ph-IAA in CD_3_OD

As the final product of this synthesis sequence, one can expect a yield of up to 4 mmol (or 1 g) of highly pure 5-Ph-IAA (Figures 5 and 6). Characterization data for 5-Ph-IAA:

^1^H NMR (400 MHz, CD_3_OD) δ 7.78 (s, 1H), 7.65–7.61 (m, 2H), 7.43–7.37 (m, 4H), 7.25 (t, J = 7.4 Hz, 1H), 7.20 (s, 1H), 3.78 (s, 2H).

^13^C NMR (101 MHz, CD_3_OD) δ 176.5, 144.2, 137.6, 133.7, 129.6, 129.2, 128.1, 127.1, 125.4, 122.2, 117.9, 112.5, 109.5, 32.0.

HRMS (ESI+) calculated m/z C_16_H_14_NO_2_ [M + H]^+^: 252.1024, found 252.1032.

The cost of raw materials required to produce this amount of 5-Ph-IAA in the lab is between one to two orders of magnitude lower than the cost of an equivalent amount of commercially available 5-Ph-IAA. Hence, the cost benefit of using lab synthesized 5-Ph-IAA would be much greater for experiments that require large quantities of the ligand, such as studies involving transcriptomic, proteomic or metabolomic comparisons.

Treatment of the *C. elegans* AID2 tester strain with 1 μM of lab-synthesized 5-Ph-IAA should be sufficient to deplete the GFP levels of this strain to levels indistinguishable from background signal (Figure 2B). Similar to commercially available 5-Ph-IAA, lab-synthesized 5-Ph-IAA also does not produce any toxicity in *C. elegans* at the ligand concentrations used for AID2 experiments. When a new batch of 5-Ph-IAA is synthesized, we recommend comparing its efficacy with that of commercially available 5-Ph-IAA or a previously used lab-synthesized batch (Figure 2B, refer to troubleshooting 4).

## Quantification and statistical analysis

^1^H NMR spectra are reported in ppm, relative to residual protonated solvent peak (CDCl_3_, 7.26 ppm). Data are reported as follows: (bs = broad singlet, s = singlet, d = doublet, t = triplet, q = quartet, m = multiplet, dd = doublet of doublets, ddd = doublet of doublet of doublets, ddt = doublet of doublet of triplets, td = triplet of doublets; coupling constant(s) in Hz; integration). Proton decoupled ^13^C NMR spectra are reported in ppm from CDCl_3_ internal standard (77.00 ppm).

Statistical comparison of GFP intensity from 1 μM 5-Ph-IAA-treated and control animals can be performed using any statistical software package (such as R or GraphPad Prism). If two groups are compared, unpaired t-test should be used. If the comparison involves more than two groups, one-way ANOVA followed by Tukey’s multiple comparisons test should be performed.

## Limitations

Though the AID technology has been used to deplete numerous proteins in a wide range of species,^1^^,^^2^^,^^3^^,^^4^^,^^5^^,^^6^^,^^7^^,^^8^ certain proteins cannot be efficiently degraded using this system.^21^ Nuclear and membrane-localized proteins have been more challenging to deplete using the AID system presumably because these proteins are less accessible to the cytoplasmic E3 ubiquitin ligase complexes.^21^^,^^22^ Several strategies have been utilized to modify the AID system for degrading these hard-to-deplete proteins and many of these strategies are also compatible with the AID2 technology (refer to troubleshooting 5).

Another limitation of the AID2 system is the inability of 5-Ph-IAA to efficiently permeate through the eggshell of *C. elegans* embryos.^11^ Using higher concentrations of 5-Ph-IAA can partially resolve this issue, but a recent study has reported that attaching an acetoxymethyl (AM) group to 5-Ph-IAA can substantially increase the eggshell permeability of this compound.^11^ Hence, experimenters trying to deplete proteins from cells that are protected by a thick layer of cuticle might consider using 5-Ph-IAA-AM, instead of 5-Ph-IAA, as the ligand for the AID2 system (refer to troubleshooting 5).

## Troubleshooting

### Problem 1

Coupling of 5-chloroindole and phenylboronic acid did not react to completion (step 8).

### Potential solution

The Suzuki coupling reaction is air sensitive due to the potential depletion of the palladium (0) catalyst by oxygen. Thoroughly degassing both solvents and reaction mixtures before starting the reaction is essential. The reaction can be carried out at 80°C for more than 1 h if needed, and NMR can be used to monitor peak consumption until completion. It is essential to ensure the reaction reaches completion, given the considerable challenge associated with separating 5-chloroindole from 5-phenylindole in the subsequent steps.

### Problem 2

The 5-Ph-IAA powder has become moist after storage at 4°C for several months (step 28).

### Potential solution

5-Ph-IAA powder can absorb moisture at 4°C if the mouth of the container is not sealed properly. If 5-Ph-IAA absorbs moisture from the environment, it would make it difficult to accurately measure the required amount of 5-Ph-IAA powder for making a stock solution. Before storing the container at 4°C, make sure that it is closed tightly and wrap the cap with a layer of parafilm to protect 5-Ph-IAA powder from external moisture.

### Problem 3

Many embryos did not hatch on the NGM-OP50 5-Ph-IAA plates (step 50).

### Potential solution

Several factors can contribute to poor survival of animals on 5-Ph-IAA plates:

• The ethanol might not have dried completely from the NGM-OP50 plates (step 33f). After addition of 5-Ph-IAA dissolved in 96% ethanol, leave the plates in the dark for at least 16 h prior to adding *C. elegans* embryos to the plate.
• Always use freshly prepared alkaline bleaching solution when synchronizing embryos (step 38). Over time, the effective sodium hypochlorite concentration decreases even if the solution is stored at 4°C. The adult worms will take longer to dissociate if the alkaline bleaching solution is old, and this will reduce the viability of the embryos that were exposed to the solution for longer. The exposure time of worms to the alkaline bleaching solution should not exceed 5 min. If it is taking longer than 5 min for the adult bodies to dissociate in the alkaline bleaching solution, change the stock of sodium hypochlorite, which might no longer have an effective 4% concentration of chlorine.

### Problem 4

A newly synthesized batch of 5-Ph-IAA does not induce effective degradation of AID∗-tagged proteins (step 57, expected outcomes).

### Potential solution

To deal with any potential batch-to-batch variability in the quality of lab-synthesized 5-Ph-IAA, every new batch should be first used with the AID2 tester strain, and a side-by-side comparison should be performed with either commercial-grade 5-Ph-IAA or a previous batch of lab-made 5-Ph-IAA that worked well in experiments. This is extremely important if a new batch of 5-Ph-IAA will be used for a critical experiment. If the new batch has a lower efficiency in depleting AID∗-tagged GFP protein in the HML1012 strain, higher doses of this batch of 5-Ph-IAA should be used to determine the concentration at which it results in similar level of AID∗-tagged GFP depletion as 1 μM of commercial-grade 5-Ph-IAA.

### Problem 5

Lab-synthesized AID2 completely degrades GFP from the AID2 tester strain but does not completely deplete the protein-of-interest in a different strain (step 69, limitations).

### Potential solution

Nuclear and transmembrane proteins are generally more difficult to deplete using the AID or AID2 systems. The following strategies can be implemented sequentially and/or in combination to improve the efficiency of degradation of AID∗-tagged endogenous proteins:

• Higher doses of 5-Ph-IAA can be used. We and others have used up to 100 μM 5-Ph-IAA without noticing any toxic effects on growth and development of *C. elegans.*^10^
• The location of the AID∗ tag can influence whether the tag is accessible to the E3 ubiquitin ligase complexes in the cytoplasm.^22^ N-terminus, C-terminus or internal tagging can be used to determine which approach yields the highest efficiency of degradation. For nuclear proteins, attaching a nuclear localization signal (NLS) to TIR1 results in more efficient degradation.^22^ Using more than one AID∗ tag can also improve depletion kinetics,^21^^,^^23^ though longer protein tags can potentially disrupt the endogenous function of proteins.
• Using the miniIAA7 (or mIAA7) degron tag, which contains a longer amino sequence from the *Arabidopsis thaliana* IAA7 protein compared to the conventional AID∗ tag, can improve the efficiency of degradation of membrane-localized proteins.^21^^,^^22^ The mIAA7 tag is compatible with both the AID and AID2 systems.^21^
• If the AID2 system is used to study protein function in *C. elegans* embryos or other cell types that are encapsulated within a thick cuticle, the 5-Ph-IAA analog with an acetoxymethyl modification can be used since it is more eggshell permeable.^11^

## Resource availability

### Lead contact

Further information and requests for resources and reagents should be directed to and will be fulfilled by the lead contact, Oliver Hobert (or38@columbia.edu).

### Technical contact

Technical questions on executing this protocol should be directed to and will be answered by the technical contacts, Oliver Hobert (or38@columbia.edu) and Makeda Tekle-Smith (mt2992@columbia.edu).

### Materials availability

This study did not generate new unique reagents.

### Data and code availability

This study did not generate any unique datasets or code.
